# Noncoding RNAs: new insights into the odontogenic differentiation of dental tissue-derived mesenchymal stem cells

**DOI:** 10.1186/s13287-019-1411-x

**Published:** 2019-09-23

**Authors:** Fuchun Fang, Kaiying Zhang, Zhao Chen, Buling Wu

**Affiliations:** 1 Department of Stomatology, Nanfang Hospital, Southern Medical University, 1838 Guangzhou Avenue North, Guangzhou, 510515 Guangdong People’s Republic of China; 2 College of Stomatology, Southern Medical University, 1838 GuangZhou Avenue North, Guangzhou, 510515 Guangdong People’s Republic of China

**Keywords:** Dental tissue, Mesenchymal stem cells, Long noncoding RNA, MicroRNA, Noncoding RNA, Odontogenic differentiation

## Abstract

Odontoblasts are cells that contribute to the formation of the dental pulp complex. The differentiation of dental tissue-derived mesenchymal stem cells into odontoblasts comprises many factors and signaling pathways. Noncoding RNAs (ncRNAs), comprising a substantial part of poly-A tail mature RNAs, are considered “transcriptional noise.” Emerging evidence has shown that ncRNAs have key functions in the differentiation of mesenchymal stem cells. In this review, we discussed two major types of ncRNAs, including microRNAs (miRNAs) and long noncoding RNAs (lncRNAs), in terms of their role in the odontogenic differentiation of dental tissue-derived stem cells. Recent findings have demonstrated important functions for miRNAs and lncRNAs in odontogenic differentiation. It is expected that ncRNAs will become promising therapeutic targets for dentin regeneration based on stem cells.

## Introduction

Mesenchymal stromal cells are derived from the mesoderm, and among these, there are stem cells (mesenchymal stem cells, MSCs) [1]. The International Society for Cellular Therapy (ISCT) (2006) proposed minimal criteria for MSCs due to the heterogeneity of isolation and cultivation procedures among different laboratories. In short, MSCs must adhere to plastic using standard culture, and express some specific cell surface markers, besides having the potential of differentiating into chondrocytes, osteocytes, and adipocytes [2]. However, these criteria are not competent to purify the homogenous MSC populations. Actually, it will produce heterogeneous, nonclonal cultures of stromal cells containing stem cells with different multipotential properties, committed progenitors, and differentiated cells when isolating MSCs according to the current criteria [3]. Hence, the definition of MSCs needs to be more standardized.

Currently, the dental tissue-derived MSCs refer to a class of cells isolated from oral tissues with MSC-like quality including the capacity for self-renewal and multilineage differentiation potential [4]. Dental tissues are specialized tissues that do not undergo continuous remodeling, and dental mesenchyme is termed “ectomesenchyme” due to its earlier interaction with the neural crest. Therefore, dental tissue-derived MSCs are derived from the neural crest, not from mesoderm [5, 6]. Oral tissues contain cells that originate from the neural crest, and among these, there are stem cells, which included human dental pulp stem cells (DPSCs) (in 2007 by Gronthos et al. [7]), periodontal ligament stem cells (PDLSCs) (in 2004 by Seo et al. [8]), stem cells from apical papillae (SCAPs) (in 2006 by Sonoyama et al. [9]), dental follicle progenitor cells (DFPCs) (in 2005 by Morsczeck et al. [10]), stem cells from exfoliated deciduous teeth (SHED) (in 2003 by Miura et al. [11]), stem cells from gingival tissue (GMSCs) (in 2009 by Zhang et al. [12] and in 2010 by Mitrano et al. [13]), MSCs from palatal connective tissue (in 2013 by Roman et al. [14]), and stem cells from alveolar bone (ABMSCs) (in 2005 by Matsubara et al. [15]). The identification of MSCs is essential for further investigation after isolation and cultivation. There are some surface markers that are generally expressed in dental tissue-derived MSCs: CD13, CD29, CD73, CD90, and CD105 [12, 13, 16–18], as shown in Table 1.

**Table 1:** Surface markers for dental tissue-derived mesenchymal stem cells

	SHED	DPSCs	SCAP	PDLSCs	DFPCs	GMSCs	MSCs from palatal tissue	ABMSCs
STRO-1	+	+	+	+	+	/	/	/
CD13	+	+	+	+	+	/	/	+
CD29	+	+	+	+	+	+	+	+
CD44	+	+	+	+	+	+	+	+
CD73	+	+	+	+	+	+	+	+
CD90	+	+	+	+	+	+	+	+
CD105	+	+	+	+	+	+	+	+
CD146	+	+	+	+	/	+	/	+
CD166	+	+	+	+	+	+	/	+

“+” indicates surface markers of cell expression; “/” indicates not reported

Odontoblasts are highly specialized cells related to the deposition and mineralization of the dentin matrix [19, 20]. They are derived from DPSCs, which originate from the neural crest. Odontoblasts contribute to the formation of the dentin pulp complex, and the process of odontogenesis is very similar to that of osteogenesis. The odontogenic activity can be stimulated in dental tissue-derived MSCs after being cultured in odontogenic medium containing dexamethasone, β-glycerophosphate, and ascorbic acid [21–27]. It is a classic and most commonly used inductive medium for odontogenic differentiation in vitro. And also, there were some other protocols such as LPS conducted for odontogenic differentiation [28–30]. Under these conditions, cells have been shown to subsequently express an osteoblast-associated gene profile, including alkaline phosphatase (ALP), collagen type 1 (COL-I), dentin matrix acid phosphoprotein 1 (DMP1), dentin sialophosphoprotein (DSPP), matrix extracellular phosphoglycoprotein (MEPE), osterix (OSX), osteocalcin (OCN), and osteopontin (OPN) [31]. Some of these genes regulate the expression of runt-related transcription factor 2 (RUNX2), OSX, and COL-I at the early stage of odontoblast differentiation, while OCN participates in the later stage of differentiation [32]. The control of odontogenic differentiation of dental tissue-derived MSCs shows great potential in the application of oral regenerative medicine and cytology treatment. Although some progress has been made in the differentiation of dental tissue-derived MSCs into odontoblasts [33–35], the precise underlying mechanisms have not been fully elucidated.

**Fig. 1:**
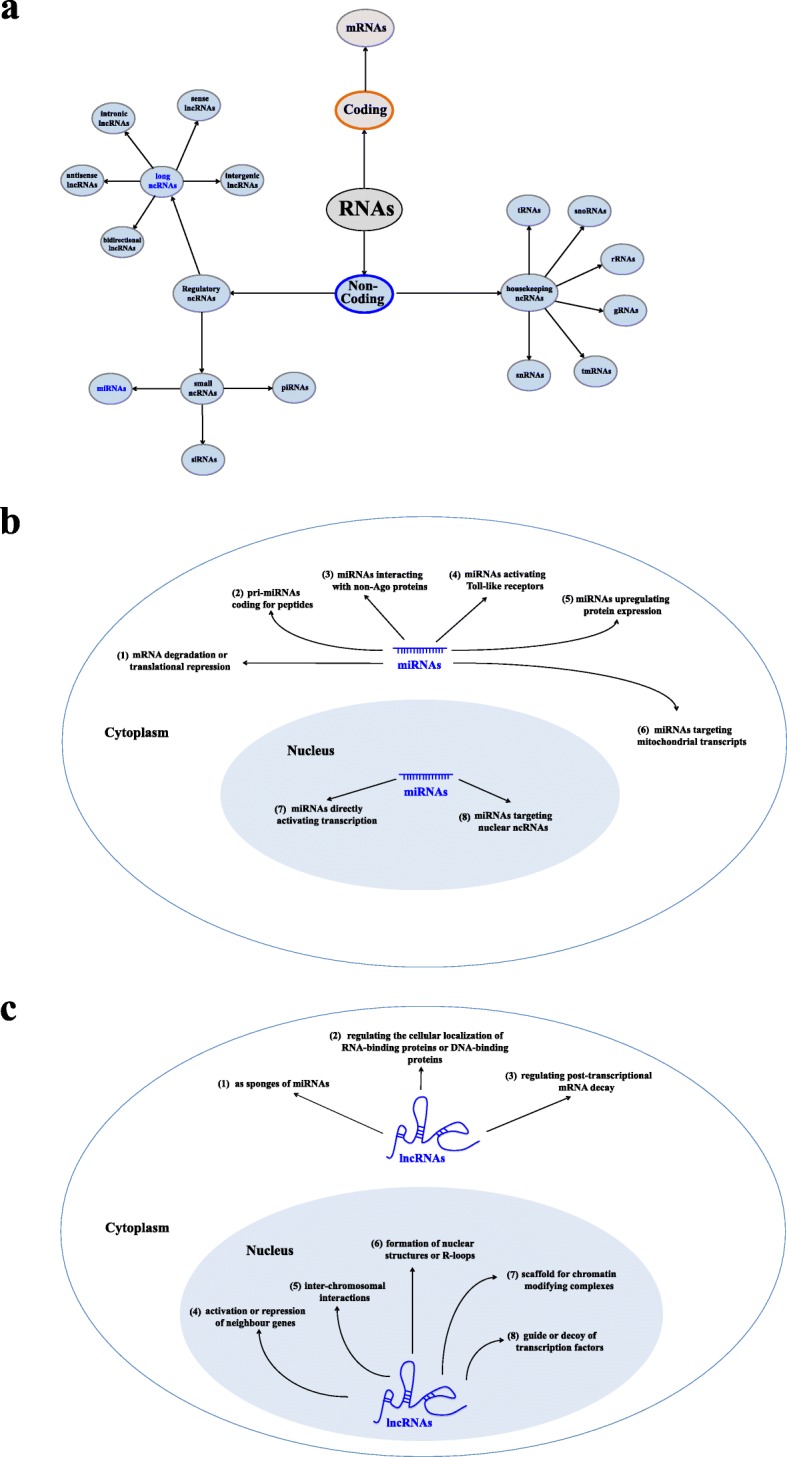
Noncoding RNA classification and functions. **a** The classification of noncoding RNAs based on their functions and length. **b** Regulatory mechanism of microRNAs. **c** Regulatory mechanism of long noncoding RNAs. rRNAs, ribosomal RNAs; tRNAs, transfer RNAs; snoRNAs, small nucleolar RNAs; snRNAs, small nuclear RNAs; tmRNAs, transfer messenger RNAs; gRNAs, guide RNAs; ncRNAs, noncoding RNAs; miRNAs, microRNAs; piRNAs, PIWI-interacting RNAs; siRNAs, small interfering RNAs; lncRNAs, long noncoding RNAs

Noncoding RNAs (ncRNAs) are a class of RNAs that do not code for proteins. Following the discovery of ncRNAs, researchers identified several ncRNAs containing short open reading frames (ORFs), which could be translated into peptides at a very low level [36]. Currently, there is no uniform standard of ncRNA classification. ncRNAs can be classified and named according to the length of the ncRNA strand, the position relationship between the ncRNAs strand and coding gene, and the function and characteristics. For example, according to subcellular localization, ncRNAs can be classified into cytoplasmic and nuclear ncRNAs. In addition, according to the difference in biological function, ncRNAs can be classified into housekeeping and regulatory ncRNAs [37, 38]. Traditionally, regulatory ncRNAs have been subjectively categorized into lncRNAs with lengths greater than 200 nt and small ncRNAs (sncRNAs) with lengths less than 200 nt. The latter can be further subcategorized into a variety of categories, including miRNAs, PIWI-interacting RNAs (piRNAs), and small interfering RNAs (siRNAs) [39], as shown in Fig. 1a. Although these ncRNAs may collectively or individually alter the cell differentiation, this review focuses on the two most important ncRNAs currently identified in odontogenic differentiation, miRNAs and lncRNAs.

### MicroRNAs analysis during odontogenesis

MiRNAs are widely present in eukaryotic cells. They are the single-strand small molecule of endogenous noncoding RNAs, and their lengths are typically 20~24 nucleotides [40]. In the canonical pathway, pri-miRNAs in the nucleus can be identified and catalyzed into pre-miRNAs by Drosha and Dicer. RNA polymerase III Dicer processes pre-miRNAs into mature miRNAs [41]. Studies have shown that mature miRNAs bind with the 3′-UTR of target mRNA completely or incompletely, which influences the stability of mRNAs or inhibits their translation and eventually downregulates protein expression [42–44]. In addition to this main mechanism, other unconventional mechanisms are gradually being explored (Fig. 1b) [45]. In the human genome, over 1000 kinds of miRNAs have been identified, and some studies have clarified that over 30% of human genes are modulated by miRNAs, which are involved in the regulation of most cellular processes [46, 47].

#### MicroRNA profiles

The main methods used to analyze miRNAs expression levels include Northern blot, microarray, high-throughput sequencing, in situ hybridization, quantitative reverse transcription polymerase chain reaction (qRT-PCR), and small RNA sequencing. Among these methods, miRNA microarrays are a high-throughput method and are the most effective [48]. We found that there were no relevant studies discussing miRNA profiles during odontogenic differentiation of dental tissue-derived mesenchymal stem cells. Microarray research conducted in 2012 by Gong et al. [49] showed that 22 miRNAs are differentially expressed after a 14-day odontogenic induction of human dental pulp cells (DPCs). Further bioinformatic analysis showed that the target genes of these miRNAs are related to the mitogen-activated protein kinase (MAPK) and the Wnt signaling pathways; both pathways are of particular interest to odontogenesis.

#### Pro-odontogenic differentiation miRNAs

miRNAs are involved in regulating transcription factors, which influence odontogenic differentiation at the transcriptional level. In 2018, Xu et al. [50] showed that the upregulated expression of miR-21 and expression of signal transducer and activator of transcription 3 (STAT3) expressions are associated with increased odontogenic differentiation in a tumor necrosis factor-α (TNF-α)-mediated odontogenesis experimental model. They showed that increasing the expression level of mature miR-21, which was able to promote phosphorylated STAT3 expression, could also be induced by upregulating p-STAT3 expression at low concentrations (1~10 ng/mL) of TNF-α. The results suggested that there is a positive reciprocal feedback loop in the miR-21/STAT3 signaling pathway that may enhance the process of odontogenic differentiation of human DPSCs.

In 2019, Huang et al. [51] showed that miR-223-3p is expressed at a higher level in inflamed pulp tissues compared with healthy tissues. miR-223-3p knockdown was shown to increase transcription of SMAD family member 3 (SMAD3), an intracellular effector of the TGF-β1 signal transduction pathway, which further inhibits odontogenic differentiation. Molecular analysis demonstrated that miR-223-3p suppresses SMAD3 transcription by dissociating from the bone morphogenetic protein 4 (BMP4) promoter 3′-UTR. These discrepancies suggested that the overexpression of miR-223-3p accelerates the odontogenic differentiation of DPSCs in an inflammatory environment by inhibiting the expression of SMAD3.

In 2014, Sun et al. [52] found that miR-34a inhibits Notch signaling to promote odontogenic differentiation of human SCAPs, whereas NOTCH activation in SCAPs inhibits cell differentiation and upregulates the expression of miR-34a. When miR-34a is overexpressed, NOTCH2 mRNA expression is downregulated, and delta-like protein 3 (DLL3), hairy and enhancer of split-1 (HES1), DSPP, RUNX2, OSX, and OCN mRNA expression is upregulated, while NOTCH2, Notch2 intracellular domain (N2ICD), and HES1 protein expression is downregulated. The opposite effects were observed when downregulating miR-34a. The study suggested that miR-34a inhibits Notch signaling by suppressing the expression of NOTCH2, N2ICD, and HES1 by directly targeting the 3′-UTR. miR-34a represses the translocation of N2ICD into the nucleus, which could suppress gene transcription by combining them, to promote the expression of related genes. In JAG1-treated SCAPs, Notch activation was shown to upregulate miR-34a transcription and suppress cell differentiation, as indicated by inhibited DSPP, ALP, RUNX2, OSX, OCN, and OPN expression. The crosstalk for miR-34a-triggered Notch repression results in cell differentiation, and activation of Notch signaling in SCAPs results in elevated miR-34a transcription that promotes cell differentiation including odontoblastic differentiation.

In addition, when the dental pulp is stimulated by trauma or infection such as pulpitis, DPSCs contained in dental pulp tissue can proliferate and migrate to the damaged area and differentiate into odontoblasts to form a restorative dentin, which can protect the dental pulp from further damage. The research conducted by Zhong et al. [53] identified differential expression of miRNAs in inflamed and healthy human dental pulps. A recent study indicated that miR-223-3p was upregulated in inflamed pulp tissues comparing with healthy ones. Further, overexpression miR-223-3p promoted odontogenic differentiation of DPSCs by targeting SMAD3. These results suggested that miR-223-3p is implicated in the regulation of odontogenic differentiation, which may be involved in the process of pulpitis repair [51]. Therefore, some miRNAs might be involved in the promotion of odontogenic differentiation of DPSCs under pulp inflammation.

#### Anti-odontogenic differentiation miRNAs

Although several miRNAs promote odontogenic differentiation of dental tissue-derived MSCs, some results from recent research have revealed miRNAs that inhibit odontogenic differentiation of dental tissue-derived MSCs, including miR-143-5p, miR-140-5p, miR-488, and hsa-let-7c.

In 2018, Zhan et al. [54] investigated the role of miR-143-5p during the odontogenic differentiation of human DPSCs. Their results suggested miR-143-5p targets RUNX2 by regulating the osteoprotegerin/receptor activator of the nuclear factor-κB ligand (OPG/RANKL) signaling pathway, which has been confirmed to be involved in odontogenesis, particularly the differentiation of dental pulp stem cells into odontoblasts. This suggests that miR-143-5p could be developed as a target of genetically modified stem cell therapy for pulp regeneration. Another study of Wang et al. [55] also identified the inhibitory role of miR-143-5p in the odontogenic differentiation of human DPSCs. They showed that the downregulated miR-143-5p expression induced the expression of the p38 MAPK signaling pathway-related gene MAPK14 and odontogenesis-related markers. The mechanism might be that downregulated miR-143-5p expression augments MAPK14 expression by inhibiting to the binding to the MAPK14 3′-UTR, activating the p38 MAPK signaling pathway to promote odontogenic differentiation of human DPSCs.

In 2017, Sun et al. [56] showed that miR-140-5p enhanced the proliferation of human DPSCs and inhibited the differentiation of human DPSCs by downregulating the expression of Toll-like receptor 4 (TLR-4) in a lipopolysaccharide (LPS)-mediated differentiation model. TLR-4 activation is significant in the progression of odontogenic differentiation promoted by LPS. Their results showed that an miR-140-5p inhibitor increased the mRNA and protein expression levels of TLR-4, while miR-140-5p mimics functioned oppositely. The decreased miR-140-5p expression level could activate TLR-4 by reducing bindings to the 3′-UTR of TLR-4 mRNA. Thus, it was concluded that during LPS-mediated odontogenic differentiation, a decreased miR-140-5p expression level could enhance TLR-4 expression and then promote odontogenic differentiation.

In 2019, Yu et al. [57] showed that a decreased miR-488 expression level enhances the odontoblastic differentiation of human DPSCs through the p38 MAPK signaling pathway by targeting MAPK1. Downregulated miR-488 expression was shown to enhance odontoblastic differentiation, likely by augmenting MAPK1 expression through decreased binding to the 3′-UTR of MAPK1 mRNA. Then, the p38 MAPK signaling pathway was activated and subsequently promoted odontogenic differentiation, as indicated by the increased expression levels of MAPK1, Ras, mitogen-activated protein kinase kinase 3/6 (MKK3/6), DSPP, ALP, and OCN.

In 2016, Ma et al. [32] showed that the insulin-like growth factor-1 (IGF-1)/IGF-1R/hsa-let-7c axis exerts a key influence on the odontogenic differentiation of IGF-1-treated SCAPs as well as the MAPK signaling pathway. IGF-1 activity is mostly facilitated through IGF-1R and is therefore known as the IGF-1/IGF-1R axis. The results indicated that IGF-1R is a potential target gene of hsa-let-7c and is negatively correlated with hsa-let-7c, both of which are upstream regulators of the MAPK pathway. JNK and p38 MAPK signaling pathways were shown to be activated by hsa-let-7c underexpression and IGF-1R overexpression then translocate into the nucleus and phosphorylate transcription factors and subsequently activate downstream odontogenic gene expression to augment odontogenic differentiation, which was indicated by the upregulated expression of several odontogenic markers in vitro. The odontogenic differentiation of IGF-1-treated SCAPs was shown to be inhibited by the IGF-1/IGF-1R/hsa-let-7c axis by suppressing the JNK and p38 MAPK signaling pathways. Additional validation of the role of the downstream signals of the MAPK pathway, especially changes in the level of transcription factors is needed.

### Long noncoding RNAs involved in odontogenesis

LncRNAs are a class of RNA molecules whose transcript length exceeds 200 nt. They do not encode proteins but regulate gene expression at various levels (epigenetic regulation, transcriptional regulation, posttranscriptional regulation, etc.) [58–60]. Initially, lncRNA was considered to be the “noise” of genomic transcription and a byproduct of RNA polymerase II transcription with no biological function. However, recent studies have shown that lncRNA is involved in many important regulatory processes, such as X chromosome silencing, genomic imprinting, chromatin modification, transcriptional activation, transcriptional interference, and intranuclear transport [61].. These regulatory roles of lncRNAs have also begun to attract wide attention. The transcripts of 4~9% of mammalian genome sequences are lncRNAs (the corresponding proportion of protein-coded RNA is 1%) [62]. Although research on lncRNA has rapidly progressed in recent years, the function of most lncRNAs remains unclear. Currently, the functions of lncRNAs cannot be speculated only from their sequences or structures. According to their positions relative to protein-coding genes in the genome, they can be divided into five types as follows: sense, antisense, bidirectional, intronic, and intergenic [63]. Thus far, more lncRNA regulatory mechanisms have been revealed (Fig. 1c).

#### Long noncoding RNA profiles

In 2016, Zheng and Jia [64] compared the profiles of freshly isolated and cultured mouse dental mesenchymal cell lncRNAs with RNA sequencing. The analysis indicated that there are a total of 144 lncRNAs (among which 108 were upregulated and 36 were downregulated) that participate in odontogenic differentiation. They also constructed 54 coexpression relationships in the odontogenic process, as well as an lncRNA-mRNA coexpression network. Further analysis showed that upregulation of maternally expressed 3 (Meg3), metastasis-associated lung adenocarcinoma transcript 1 (Malat1), X-inactive specific transcript (Xist), distal-less homeobox 1, antisense (Dlx1as) expression is associated with the promotion of the odontogenic process. Moreover, Dlx1as, which is negatively correlated with Dlx1, acts as a positive modulator in the odontogenic process of dental mesenchymal cells. Their results suggested that the dysregulation of lncRNAs is associated with the loss of odontogenic potential in mouse dental mesenchymal cells. In 2016, Chen et al. [65] used lncRNA microarray profiling to examine the lncRNA expression during the odontogenic differentiation of human dental pulp cells (DPCs). A total of 139 lncRNAs with a greater than twofold change were shown to be dysregulated in the 14-day induction group compared with the noninduced control group. Among these lncRNAs, 67 were upregulated while 72 were downregulated. Pathway analysis was used to reveal the biological functions of lncRNAs with their target genes, in which the cell cycle, extracellular matrix receptor interaction, and transforming growth factor-β (TGF-β) signaling pathways were implicated. These results indicate that lncRNAs might play crucial roles in this process and regulate odontogenesis-related pathways. Further functional analysis of these lncRNAs is needed to provide conclusive evidence supporting an underlying regulatory mechanism during odontogenesis.

#### H19

Notably, lncRNA H19 is a highly conserved imprinted gene that encodes an ~ 2.6-kb polyadenylated lncRNA and exerts a variety of functional activities both in the nucleus and in the cytoplasm [66]. H19 has many different biological functions including regulatory roles in cell proliferation and differentiation and in cancer as oncogene and tumor suppressor gene [67–69]. In addition, H19 is both epigenetically regulated and utilizes epigenetic mechanisms to regulate the odontogenic differentiation of human DPSCs. In 2018, Zeng et al. [70] demonstrated that overexpression of H19 could decrease the expression level of *S*-adenosylhomocysteine hydrolase (SAHH), which is the only enzyme to catalyze *S*-adenosylhomocysteine (SAH) into homocysteine in humans. The decreased expression level of SAHH was shown to reduce the expression level of SAH, which can block the methylation activity of DNMTs. Thus, H19, along with the downregulated SAHH, could repress the activity of DNA methyltransferase 3B (DNMT3B). Upregulated H19 expression significantly repressed SAHH and DNMT3B activities, which then enhanced the DLX3 expression by inhibiting the DNMT3B-medicated methylation of DLX3. Additionally, H19 overexpression reduced the expression levels of DSPP, DMP-1, ALP, Nes, DLX3, and DLX5, whereas the opposite effect was observed when H19 was downregulated. Therefore, the H19/SAHH axis epigenetically promotes the odontogenic differentiation of human DPSCs. In a recent study [71], miR-675 was shown to promote the odontogenic differentiation of human DPCs by inhibiting the DNMT3B-mediated methylation of DLX3. Therefore, we speculate that H19 and miR-675, which are two related ncRNAs, are involved in odontogenic differentiation. More studies are needed to investigate the regulatory mechanism of H19/miR-675 axis during odontogenic differentiation.

What is more, Li et al. [72] reported that overexpression of H19 led to the enhanced odontogenesis of SCAPs, whereas knockdown of H19 inhibited these effects. Further mechanical study showed that H19 bounded to miR-141 as competing endogenous RNA (ceRNA) and consequently led to increasing SPAG9, which is important in the activation of p38 and JNK MPAK signaling pathways through significantly elevating phosphorylated levels of p38 and JNK. This study revealed that lncRNA-H19/miR-141/SPAG9 axis modulates the odontogenic differentiation of SCAPs via MAPK pathways.

#### DANCR

In 2012, Kretz et al. [73] identified a lncRNA, which was downregulated during stem cell differentiation and required to maintain epidermal stem cells and osteoblast cells in an undifferentiated cell state. This lncRNA was named anti-differentiation noncoding RNA (ANCR, subsequently named differentiation antagonizing nonprotein coding RNA (DANCR)). Based on previous studies, Chen et al. [65] reported that DANCR exerts negative effects on the differentiation of human DPCs into odontoblast-like cells. Based on molecular mechanisms, the expression level of β-catenin and the phosphorylation level of GSK-3β were decreased in DANCR-overexpressing DPCs. The inhibition of GSK-3β was shown to contribute to the translocation of β-catenin into the nucleus, where it combines some transcriptional factors to affect the expression of DSPP and DMP-1. It was indicated that DANCR cause subsequent suppression of the Wnt/β-catenin signaling pathway and odontoblastic differentiation. As a result, DANCR might act as an important modulator of the odontoblast-like differentiation of human DPCs.

## Conclusions

**Fig. 2:**
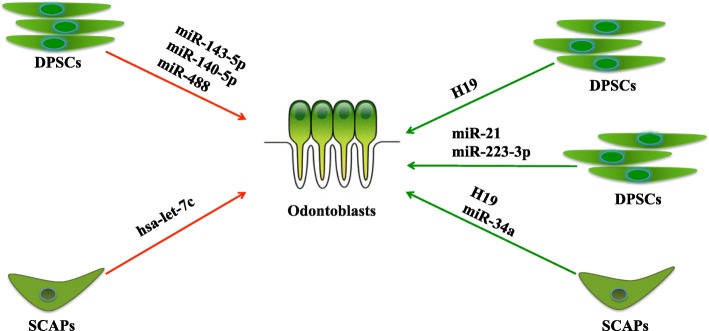
Reported ncRNAs that regulate the odontogenic differentiation of dental tissue-derived stem cells. Green line, promotion; red line, inhibition. DPSCs, dental pulp stem cells; H19, imprinted maternally expressed transcript; SCAPs, stem cells from apical papillae

In summary, numerous ncRNAs are involved in the odontogenic differentiation of dental tissue-derived stem cells (Fig. 2). ncRNAs offer an exciting avenue of odontogenesis-related gene regulation that has not yet been fully explored. With the discovery of miRNAs and lncRNAs involved in this process, it could be possible to use these ncRNA-based therapeutic strategies in the field of dental pulp regeneration and repair.

**Fig. 3:**
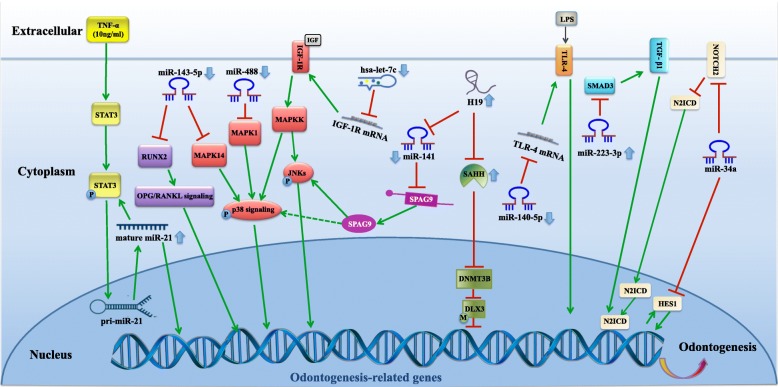
The regulating mechanisms of ncRNAs that contribute to the odontogenic differentiation of dental tissue-derived stem cells. The green arrow indicates promotion, and the red T indicates inhibition. ALP, alkaline phosphatase; BMP4, bone morphogenetic protein 4; COL-I, collagen type 1; DLX3, distal-less homeobox 3; DNMT3B, DNA methyltransferase 3B; DMP1, dentin matrix acid phosphoprotein 1; DSPP, dentin sialophosphoprotein; H19, imprinted maternally expressed transcript; HES, hairy/enhancer of split; IGF-1, insulin-like growth factor-1; JNK, c-Jun N-terminal kinase; LPS, lipopolysaccharide; MAPKK, mitogen-activated protein kinase kinase; N2ICD, Notch2 intracellular domain; NICD, Notch intracellular domain; OCN, osteocalcin; OPG/RANKL, osteoprotegerin/receptor activator of the nuclear factor-κB ligand; OPN, osteopontin; OSX, osterix; RUNX2, runt-related transcription factor 2; SAHH, *S*-adenosylhomocysteine hydrolase; SMAD3, SMAD family member 3; SPAG9, sperm-associated antigen 9; STAT3, signal transducer and activator of transcription 3; TGF-β, transforming growth factor-β; TNF-α, tumor necrosis factor-α; TLR-4, Toll-like receptor 4

Based on previous studies, the research of ncRNAs during odontogenic differentiation of dental tissue-derived stem cells is mainly focused on miRNAs. The demonstrated mechanism includes the inhibition of target gene mRNA (miR-143-5p, miR-488, miR-223-3p, miR-34a, hsa-let-7, and miR-140-5p) and upregulation protein expression (miR-21). Other unconventional regulating mechanisms might have a potential function during odontogenic differentiation. Currently, miRNAs are considered as the strongest therapeutic potential tool due to the clear functioning mode and pleiotropic mechanism of action. miRNA-based therapy could be a valuable tool to promote pulp regeneration and repair in a comprehensive and sophisticated way. There are fewer studies concerning lncRNAs during this process. Among these, H19 and DANCR are two well-known lncRNAs. H19 epigenetically regulates odontogenic differentiation through the methylation of target genes while DANCR epigenetically regulates differentiation through the Wnt/β-catenin signaling pathway (Fig. 3 and Table 2). Other types of ncRNAs deserve further exploration.

**Table 2:** Noncoding RNAs involved in the odontogenic differentiation of dental tissue-derived mesenchymal stem cells

ncRNA	Gene ID	Effects	Modes of action	Associated targets or pathways	Cell category	References
lncRNA	H19 (imprinted maternally expressed transcript)	Promotes odontogenic differentiation	(1) H19/SAHH axis	DNMT3B decreases and DLX3 increases	Human DPSCs	Zeng et al. 2018 [70, 71]
			(2) H19/miR-141/SPAG9 axis	p38 and JNK MAPK pathway	Human SCAPs	Li et al. 2019 [72]
lncRNA	DANCR (differentiation antagonizing nonprotein coding RNA)	Blocks odontoblast-like differentiation	GSK-3β and β-catenin suppression	Canonical Wnt/β-catenin signaling pathway	Human DPCs	Chen et al. 2016 [65]
miRNA	miR-21	Positively modulates odontoblastic differentiation	(1) Increasing p-STAT3	A positive feedback loop in the miR-21/STAT3 signaling pathway	Human DPSCs	Xu et al. 2018 [50]
			(2) Increased by p-STAT3			
miRNA	miR-143-5p	Inhibits the differentiation of human DPSCs into odontoblasts	(1) Interacting with RUNX2 3′-UTR	(1) RUNX2 suppression OPG/RANKL signaling pathway	Human DPSCs	Zhan et al. 2018 [54]
			(2) Interacting with the MAPK14 3′-UTR	(2) MAPK14 suppression p38 MAPK signaling pathway		Wang et al. 2019 [55]
miRNA	miR-140-5p	Inhibits odontogenic differentiation	Interacting with the TLR-4 3′-UTR	LPS/TLR-4 signaling pathway	Human DPSCs	Sun et al. 2017 [56]
miRNA	miR-223-3p	Promotes odontoblastic differentiation	Interacting with the BMP4 3′-UTR	SMAD3 suppression TGF-β1 signal transduction pathway	Human DPSCs	Huang et al. 2019 [51]
miRNA	miR-448	Blocks odontogenic differentiation	Interacting with the MAPK1 3′-UTR	p38 MAPK signaling pathway	Human DPSCs	Yu et al. 2019 [57]
miRNA	hsa-let-7c	Inhibits the odontogenic differentiation of IGF-1-treated human SCAPs	IGF-1/IGF-1R/hsa-let-7c axis	JNK and p38 MAPK signaling pathways	Human SCAPs	Ma et al. 2016 [32]
miRNA	miR-34a	Promotes odontogenic differentiation	(1) Interacting with NOTCH2 and HES1 3′-UTR	Crosstalk between miR-34a and Notch signaling	Human SCAPs	Sun et al. 2014 [52]
			(2) Activated by Notch signaling			

*ALP* alkaline phosphatase, *BMP4* bone morphogenetic protein 4, *COL-I* collagen type 1, *DLX3* distal-less homeobox 3, *DNMT3B* DNA methyltransferase 3B, *DMP1* dentin matrix acid phosphoprotein 1, *DSPP* dentin sialophosphoprotein, *GSK-3β* glycogen synthase kinase 3, *HES* hairy/enhancer of split, *IGF-1* insulin-like growth factor-1, *JNK* c-Jun N-terminal kinase, *LPS* lipopolysaccharide, *MAPK* mitogen-activated protein kinase, *N2ICD* Notch2 intracellular domain, *NICD* Notch intracellular domain, *OCN* osteocalcin, *OPG/RANKL* osteoprotegerin/receptor activator of the nuclear factor-κB ligand, *OPN* osteopontin, *OSX* osterix, *RUNX2* runt-related transcription factor 2, *SAHH S*-adenosylhomocysteine hydrolase, *SMAD3* SMAD family member 3, *SPAG9* sperm-associated antigen 9, *STAT3* signal transducer and activator of transcription 3, *TGF-β* transforming growth factor-β, *TNF-α* tumor necrosis factor-α, *TLR-4* Toll-like receptor 4

With the advent of high-throughput sequencing and next-generation microarrays, novel ncRNAs with regulatory functions can be discovered more quickly and accurately based on the bioinformatics database prediction. Currently, conventional methods, including overexpression/inhibition, luciferase reporting, qRT-PCR, and Western blot, are utilized to explore the regulatory mechanism. However, some new methods have emerged, including CRISPR/Cas9; RIP; chromatin isolation by RNA purification (ChIRP); RNA pull-down; cross-linking immunoprecipitation (CLIP); cross-linking, ligation, and sequencing of hybrids (CLASH); and capture hybridization analysis of RNA targets (CHART), which can also be combined with mass spectrometry technology. The emergence of these new technologies provides an ideal research platform for elucidating the binding mechanism of specific proteins. In addition, research on the mechanisms of miRNAs is mainly focused on the inhibition of target genes to regulate odontogenic differentiation. However, there are few studies on the nonconventional mechanism mentioned in these studies. Fewer studies focusing on lncRNAs have been conducted. Interest in the contribution of ncRNAs to the odontogenesis of dental tissue-derived mesenchymal stem cells is flourishing, but more effort is currently required to determine the full extent of this contribution and the mechanisms by which ncRNAs exert their potential effects.

## Data Availability

Not applicable.

## Abbreviations

ABMSCs: Stem cells from alveolar bone
ALP: Alkaline phosphatase
BMP4: Bone morphogenetic protein 4
COL-I: Collagen type 1
DFPCs: Dental follicle progenitor cells
DLL3: Delta-like protein 3
DLX3: Distal-less homeobox 3
DMP1: Dentin matrix acid phosphoprotein 1
DNMT3B: DNA methyltransferase 3B
DPCs: Dental pulp cells
DPSCs: Dental pulp stem cells
DSPP: Dentin sialophosphoprotein
GMSCs: Stem cells from gingival tissue
gRNAs: Guide RNAs
H19: Imprinted maternally expressed transcript
HES: Hairy/enhancer of split
IGF-1: Insulin-like growth factor-1
JNK: c-Jun N-terminal kinase
lncRNAs: Long noncoding RNAs
LPS: Lipopolysaccharide
MAPKK: Mitogen-activated protein kinase kinase
MEPE: Matrix extracellular phosphoglycoprotein
miRNAs: MicroRNAs
MSCs: Mesenchymal stem cells
N2ICD: Notch2 intracellular domain
ncRNAs: Noncoding RNAs
NICD: Notch intracellular domain
OCN: Osteocalcin
OPG/RANKL: Osteoprotegerin/receptor activator of the nuclear factor-κB ligand
OPN: Osteopontin
OSX: Osterix
PDLSCs: Periodontal ligament stem cells
piRNAs: PIWI-interacting RNAs
rRNAs: Ribosomal RNAs
RUNX2: Runt-related transcription factor 2
SAHH: *S*-adenosylhomocysteine hydrolase
SCAPs: Stem cells from apical papillae
SHED: Stem cells from exfoliated deciduous teeth
siRNAs: Small interfering RNAs
SMAD3: SMAD family member 3
snoRNAs: Small nucleolar RNAs
snRNAs: Small nuclear RNAs
SPAG9: Sperm-associated antigen 9
STAT3: Signal transducer and activator of transcription 3
TGF-β: Transforming gro wth factor-β
TLR-4: Toll-like receptor 4
tmRNAs: Transfer messenger RNAs
TNF-α: Tumor necrosis factor-α
tRNAs: Transfer RNAs
